# Investigation on synthesis and properties of isosorbide based bis-GMA analogue

**DOI:** 10.1007/s10856-012-4594-6

**Published:** 2012-03-10

**Authors:** Jan Łukaszczyk, Bartosz Janicki, Achim Frick

**Affiliations:** 1 Faculty of Chemistry, Department of Physical Chemistry and Technology of Polymers, Silesian University of Technology, ul. M. Strzody 9, 44-100 Gliwice, Poland; 2 Institute of Polymer Science and Processing (iPSP), Aalen University, Beethoven str. 1, 73430 Aalen, Germany

## Abstract

The aim of this work was to synthesize and investigate properties of a novel dimethacrylic monomer based on bioderived alicyclic diol—isosorbide. Its potential as a possible substitute of 2,2-bis[4-(2-hydroxy-3-methacryloyloxypropoxy)phenyl]propane (BISGMA), widely used in dental restorative materials and suspected for toxicity was assessed. The novel monomer was obtained in a three-step synthesis. First, isosorbide was etherified by a Williamson nucleophilic substitution and subsequently oxidized to isosorbide diglycidyl ether (ISDGE). A triphenyl phosphine catalyzed addition of methacrylic acid to ISDGE resulted in 2,5-bis(2-hydroxy-3-methacryloyloxypropoxy)- 1,4:3,6-dianhydro-sorbitol (ISDGMA). The monomer obtained was photopolymerized using camphorquinone/2-(dimethylamino)ethyl methacrylate initiating system. Next, compositions with triethylene glycol dimethacrylate (TEGDMA) were prepared and polymerized. Double bond conversion, polymerization shrinkage and water sorption of resulting polymers were determined. Selected mechanical (flexular strength and modulus, Brinell hardness) and thermomechanical (DMA analysis) properties were also investigated. BISGMA based materials were prepared as reference for comparison of particular properties.

## Introduction

Current dental composites comprise three basic components: highly crosslinked polymeric matrix, inorganic filler and coupling agent used to ensure good matrix-filler adhesion. The organic matrix is formed as three-dimensional network resulting from free-radical photopolymerization of bulky dimethacrylic monomers. Use of such monomers provides lower polymerization shrinkage, higher chemical resistance and reduces leaching of unreacted monomer that has not been built into the matrix. Most of the composite resins widely used in restorative dental materials contain bisphenol A based 2,2-bis[4-(2-hydroxy-3-methacryloyloxypropoxy)phenyl]propane (BISGMA) and low viscosity monomers, used as diluents such as triethylene glycol dimethacrylate (TEGDMA) and 1,6-bis-(methacryloyloxy-2-ethoxycarbonylamino-2,4,4-trimethylhexane (UDMA) [1, 2]. Although being very popular, BISGMA resin introduced in 1962 by Bowen [3] has relatively high viscosity which limits the amount of filler that can be introduced into the composite. The use of low viscosity co-monomer like TEGDMA helps to overcome this problem but adversely affect the properties of matrix by increasing the water sorption and polymerization shrinkage [4]. Recent literature describes that monomers like BISGMA and TEGDMA are health uncertain and may even induce apoptosis of human dental pulp. This effect occurs immediately after the contact of the composition with human tissue just before curing as well as after a long term release of unreacted monomers [5–11].

The development of new monomers for restorative filling materials is predominantly motivated by need to overcome the main shortcomings of resin composites, i.e., relatively high water sorption, polymerization shrinkage, high viscosity, cytotoxicity and residual monomer release. The current research is mainly focused on synthesis of new high molecular weight dimethacrylic monomers as well as multimethacrylates of a dendritic structure [12–21]. Efforts to improve performance of restorative materials led also to the development of innovative monomers for ring-opening polymerisation—siloranes [22–24].

In this work isosorbide based BISGMA analogue was considered as a possible dimethacrylic monomer for dental and bone tissue repair applications. Isosorbide is bioderived and biodegradable diol qualified by Food and Drug Administration as “generally recognized as safe” (GRAS) [25]. It is produced by the double dehydration of sorbitol obtained in two step process from starch. Isosorbide—a V-shaped bicyclic diol is composed of two fused tetrahydrofuran (THF) rings. This structure provides high stiffness of its molecule and good chemical resistance [26]. Many isosorbide derivatives have already found an application in pharmaceutical industry and in cosmetics [27]. Those features make this “green” material an interesting candidate for replacing health uncertain bisphenol A in the synthesis of dental dimethacrylic monomers.

Obtained in three-step synthesis (2,5-bis(2-hydroxy-3-methacryloyloxypropoxy)-1,4:3,6-dianhydro-sorbitol (ISDGMA) was characterized by means of NMR, IR spectroscopy and ESI–MS spectrometry. Subsequently ISDGMA was photopolymerized using typical initiating system comprising of camphorquinone (CQ) and 2-(dimethylamino)ethyl methacrylate (DMAEMA). Additionally composition of ISDGMA:TEGDMA 60:40 [w/w] was prepared and cured. Thermal and mechanical properties as well as water sorption and double bond conversion of resulting materials were determined and compared with selected dimethacrylates presently used in dental applications.

## Materials and methods

### Materials and instruments

Isosorbide (IS, 98%), methacrylic acid (MAA, 99%, stabilized), phenothiazine (>99%), bisphenol A diglycidyl ether (BADGE, Epoxy eq. 172 g/eq.), camphorquinone (CQ, 97%), triethylene glycol dimethacrylate (95%, stabilized) all from Sigma-Aldrich. Allyl bromide (AllBr, 99%, stabilized), tetrabutylammonium bromide (TBAB, >99%), *m*-chloroperoxybenzoic acid (MCPBA, 75%), triphenylphosphine (TPhP, 99%), 2-(dimethylamino)ethyl methacrylate (DMAEMA, 99%, stabilized) all from Acros Organics. 2,2-Dimethoxy-2-phenylacetophenone (Irgacure 651) from Ciba. Dichloromethane (pure) and potassium carbonate (pure) from POCH. Magnesium sulphate (pure), and KOH (pure for analysis) from Stanlab. Phosphate buffer solution (PBS, pH = 7.41) from LabStand. All substances were used as received.

NMR spectra were recorded with UNITY/INOVA (Varian) spectrometer operating at 300 MHz (^1^H NMR) and at 75 MHz (^13^C NMR). CDCl_3_ and tetramethylsilane (TMS) were used as a solvent and internal standard, respectively. The IR spectra were recorded with BIORAD FTS 175L spectrophotometer at room temperature after applying a thin film on a KBr disk. ESI-MS analysis was carried out on Ionspec 4.7 T Ultima FTMS FT-ICR MS Fourier Transform Ion Cyclotron Resonance Mass Spectrometer with ESI TOF ionisation.

Viscosities of synthesized monomers were measured at 25 and 40°C by means of Malvern Instruments Gemini 200 plate-plate rheometer, equipped with steel plates (*ϕ* = 25 mm) and 1 mm gap between them. The density of monomers and polymers were determined using the liquid pyknometer at 25°C according to PN-EN ISO 1183-1:2006. The epoxy number (EN) of the product was determined by titration of a sample dissolved in HCl/dioxane solution, with ethanolic KOH solution according to PN87/C-89085/13. Phenolphthalein was used as an indicator. The acid number (AN) during reactions was monitored by titration with an alcoholic KOH solution using phenolphthalein as an indicator according to PN-87/C-89082/11.

### Synthesis of dimethacrylic monomers

#### Synthesis of BISGMA

The BISGMA resin was synthesized by reacting diglycidyl ether of bisphenol A, with methacrylic acid using triphenylphosfine as a catalyst. A ratio of 1.25/1 equivalents of acid to DGEBA was used together with 0.3% of catalyst and 1,000 ppm of hydroquinone to avoid the thermal polymerization of the vinyl groups. The reaction was carried out in a round-bottom flask with temperature control and nitrogen purge. The temperature was maintained at 75°C during the first hour of the reaction to avoid rapid increase of temperature due to the high initial reaction rate, and then it was raised and kept at 100°C until a final conversion of 99% was reached. The conversion of acid groups was monitored by titration with an alcoholic KOH solution in presence of phenolphthalein. The excess of methacrylic acid was washed out from the synthesis product with distilled water. The remaining water was removed under vacuum at 50°C. The final product was stabilized with 300 ppm of hydroquinone (97% yield).

#### Synthesis of ISDGMA

##### *I step*: preparation of isosorbide diallyl ether (IS-All)

IS-All was prepared by Williamson etherification reaction. Isosorbide was dissolved in a 50% aqueous KOH. Allyl bromide was used as an alkylating agent together with phase transfer catalyst (tetrabutylammonium bromide). Reagents were used at ratio of 1 mol of isosorbide, 6 mol of allyl bromide, 6 mol of KOH and 0.06 mol of TBAB. The product obtained after heating under reflux for 7 h was purified by vacuum distillation collecting fraction boiling at 108°C/1 Torr (96% yield). The results of spectroscopic analysis are as follows: FT-IR (cm^−1^): *ν*_=C–H_ = 3,079, *ν*_C=C_ = 1,647. ^1^H NMR (*δ*, ppm): 5.9 (1H, allyl –CH), 5.8 (1H, allyl –CH′), 5.2/5.2 (1H, allyl =CH_2_ *Z*), 5.1/5.1 (1H, allyl =CH_2_ *E*), 4.5 (1H, –CH isos), 4.4 (1H, –CH isos), 4.1 (1H, allyl –OCH_2_), 3.9–4.0 (7H, 3× allyl -OCH_2_, 2× –CH isos, –CH_2_ isos), 3.8 (1H, –CH_2_ isos), 3.5 (1H, –CH_2_ isos). ^13^C NMR (*δ,* ppm): 134.9/134.6 (allyl –CH), 118.1/117.8 (allyl =CH_2_), 86.7 (–CH isos), 84.2 (–CH isos), 80.6 (–CH isos), 79.8 (–CH isos), 73.8 (–CH_2_ isos), 72.0 (allyl –OCH_2_), 70.9 (–CH_2_ isos), 70.2 (–CH_2_ isos).

##### *II step:* preparation of isosorbide diglycidyl ether (ISDGE)

ISDGE was obtained by epoxidation of IS-All at room temperature. Substrate was dissolved in CH_2_Cl_2_ and stirred for 24 h with oxidizing agent (*m*-chloroperoxybenzoic acid). Reagents were used at ratio of 1 mol of IS-All and 2.2 mol of MCPBA. After completion of the reaction precipitated *m*-chlorobenzoic acid was separated and anhydrous K_2_CO_3_ added in order to remove all acidic residue. Resulting slurry was subsequently filtrated and the solvent removed under vacuum (91% yield). Epoxy number of product obtained was equal to 0.76 mol/100 g (teor. 0.77 mol/100 g). The results of spectroscopic analysis are as follows: FT-IR (cm^−1^): *ν*_C–O–C oxir_ = 1,257, *ν*_C–O–C isos_ = 1,093, *ν*_C–O–C oxir_ = 910, *ν*_C–O–C oxir_ = 854. ^1^H NMR (*δ,* ppm): 4.7 (1H, –CH isos), 4.5 (1H, –CH isos), 3.9–4.2 (4H, –CH_2_ isos, 2× –CH isos), 3.8–3.9 (1H, –CH_2_ isos), 3.6 (2H, –CH_2_ oxir), 3.3–3.5 (3H, –CH_2_ isos, –CH_2_ oxir), 3.2 (2H, 2× –CH oxir), 2.8 (2H, 2× –CH_2_ oxir), 2.6 (2H, 2× –CH_2_ oxir). ^13^C NMR (*δ,* ppm): 85.9 (–CH isos), 84.5 (–CH isos), 80.4 (–CH isos), 80.1 (–CH isos), 72.9 (–CH_2_ isos), 71.5 (–CH_2_ isos), 70.8 (–OCH_2_ oxir), 69.8 (–OCH_2_ oxir), 50.3 (–CH oxir), 44.0 (–CH_2_ oxir).

##### *III step:* preparation of BISGMA isosorbide analogue (ISDGMA)

ISDGMA was synthesized via catalytic addition of methacrylic acid in the same conditions as described in “Synthesis of BISGMA” section. After completion of the reaction the raw product was dissolved in CH_2_Cl_2_ and K_2_CO_3_ was added in order to remove residual methacrylic acid. Precipitated salt was filtrated off and solvent removed under vacuum at 40°C (92% yield). 300 ppm of hydroquinone was added to prevent spontaneous polymerization during storage. The results of spectroscopic analysis are as follows: ESI-MS: 431.6 [M + H^+^], 448.6 [M + NH_4_^+^], 453.5 [M + Na^+^], 469.3 [M + K^+^], FT-IR (cm^−1^): *ν*_O–H_ = 3,050–3,600, *ν*_C–H_ = 2,800–3,000, *ν*_C=O_ = 1,710, *ν*_C=C_ = 1,640, *ν*_C–O–C_ = 1,173, *ν*_C–O–C_ = 1,091. ^1^H NMR (*δ*, ppm): 6.1/5.6 (4H, =CH_2_), 4.7 (1H, –CH isos), 4.5 (1H, –CH isos), 4.2 (4H, –CH_2_), 3.8–4.1 (5H, 2× –CH isos, 3× –CH_2_ isos), 3.5–3.8 (5H, 3× –CH_2_, –CH_2_ isos, –OH), 1.9 (1H, –CH_3_). ^13^C NMR (*δ,* ppm): 167.3 (C=O), 135.8 (C=C), 126.0 (C=C), 86.0 (–CH isos), 84.7 (–CH isos), 80.7 (–CH isos), 80.2 (–CH), 80.0 (–CH), 73.0 (–CH_2_ isos), 72.2 (–CH_2_), 71.8 (–CH_2_), 70.4 (–CH isos), 69.0 (–CH_2_), 68.6 (–CH_2_), 65.3 (–CH_2_ isos), 18.2 (–CH_3_).

### Polymerization

Composition of BISGMA or ISDGMA with TEGDMA in a ratio of 60:40 [w/w] was prepared in a glass vessel by stirring at 40°C for 30 min. Resulting mixtures as well as single monomers were afterwards mixed with 0.4% of CQ and 1% of DMAEMA and poured into square-shape aluminum moulds with glass bottom in amount assuring desired thickness of resulting specimens (4 mm). Photopolymerization was carried out under a mercury lamp (FAMED-1, model L-6/58), 375 W) set 15 cm above the mould for 30 min. for each sample side. Afterwards, the inhibition layer of the specimen was removed by polishing with fine sandpaper 1,000 grid.

### Mechanical properties

The effect of temperature on viscoelastic properties of the obtained polymers in the limits of linear dependence between stress and strain was determined using a GABO Qualimeter Eplexor 150 N DMA device equipped with a testing needle of ϕ = 1 mm, working in compression mode. Thermomechanical properties of the cured resins were evaluated from complex modulus (E*) and tangent δ curves obtained at constant frequency (10 Hz). Measurements for all 1 mm thick samples were made in the temperature range 25–250°C at a constant heating rate of 5°C/min. The flexural strength and the flexural modulus were determined in accordance with ISO 178:2003 in a three-point bending test, using a universal testing machine Zwick/Roell Z005 equiped with 5 kN crosshead. Specimen dimensions were: 10 ± 0.2 mm × 4 ± 0.2 mm × 83 mm. Measurements were carried out at room temperature with a crosshead speed of 5 mm/min. The ball indentation hardness (Brinell hardness) was determined in accordance with ISO 2039-1:2004 using VEB Werkstoffprüfmaschinen Leipzig 300/250 apparatus. Every measurement was repeated five times for each investigated material.

### Determination of the degree of double bonds conversion

Compositions of monomers were prepared in the same manner as in 2.3, later 1% [w/w] of Irgacure—651 which is an UV initiator was added either into monomers mixture or into pure monomers. Approximately 7 mg samples were cured in aluminum pans in a nitrogen atmosphere. The isothermal photopolymerization study by means of photo-DSC was performed at a temperature of 40°C which is close to human oral temperature using a METTLER-TOLEDO, DSC822e calorimeter. It was equipped with a Hamamatsu Lightning Cure LC8 (Hg–Xe lamp) with two beams, one for the sample and the other for the reference. Two scans were performed on each sample in order to subtract the thermal effect of the UV irradiation on the sample from the photocuring experiment. Each method consisted of 4 min of temperature conditioning, 10 min of irradiation and finally 4 min more without UV light. The light intensity at the sample pan position was measured by the carbon black method as 68.1 mW cm^−2^. Degree of double bonds conversion (DC) was calculated according to the following equation:

$$ {\text{DC}}(\% ) = \frac{{\Updelta H_{x} }}{{\Updelta H_{o} n}} \cdot 100 $$

where *ΔH*_*x*_ is the curing enthalpy of the sample, *ΔH*_*o*_ is the theoretical molar polymerization enthalpy of methacrylic double bond (57 kJ/mol) and *n* is the number of moles of methacrylic groups present in the sample. Measurements were repeated five times for each monomer and composition.

### Determination of the polymerization shrinkage

The polymerization shrinkage (PS) was determined by measuring densities (as described in “Materials and instruments” section) of monomers and monomers mixtures and comparing them with densities of resulting polymers. The following equation was used for calculations:

$$ PS(\% ) = \frac{{d_{p} - d_{m} }}{{d_{p} }} \cdot 100 $$

where *d*_*p*_ is the density of polymer and *d*_*m*_ is the density of monomer/monomers mixture.

### Measurement of the water sorption

In order to determine the water sorption of polymerized materials 5 disk-like samples (15 mm × 1 mm) of each material were prepared according to EN-ISO 4049:2009. Immediately after polymerization, the specimens were placed in a pre-conditioning oven at 37°C. The specimens were repeatedly weighed until constant mass (*m*_o_) was obtained. They were then individually placed in sealed glass vessels containing 10 ml of PBS at 37°C. After 7 days, vessels were removed from the oven and left at room temperature for 1 h. The specimens were gently wiped with a soft absorbent paper and weighed (*m*_7_).

Water sorption (WS) was calculated using the following formula:

$$\text{WS}(\upmu {\text{g}}/{\text{mm}}^{3}) = \frac{{m_{7} -
m_{o} }}{V}$$

where *m*_*o*_ is the weight before immersing, *m*_*7*_ is weight after 7 days and *V* is an initial volume of the sample.

## Results and discussion

**Fig. 1:**
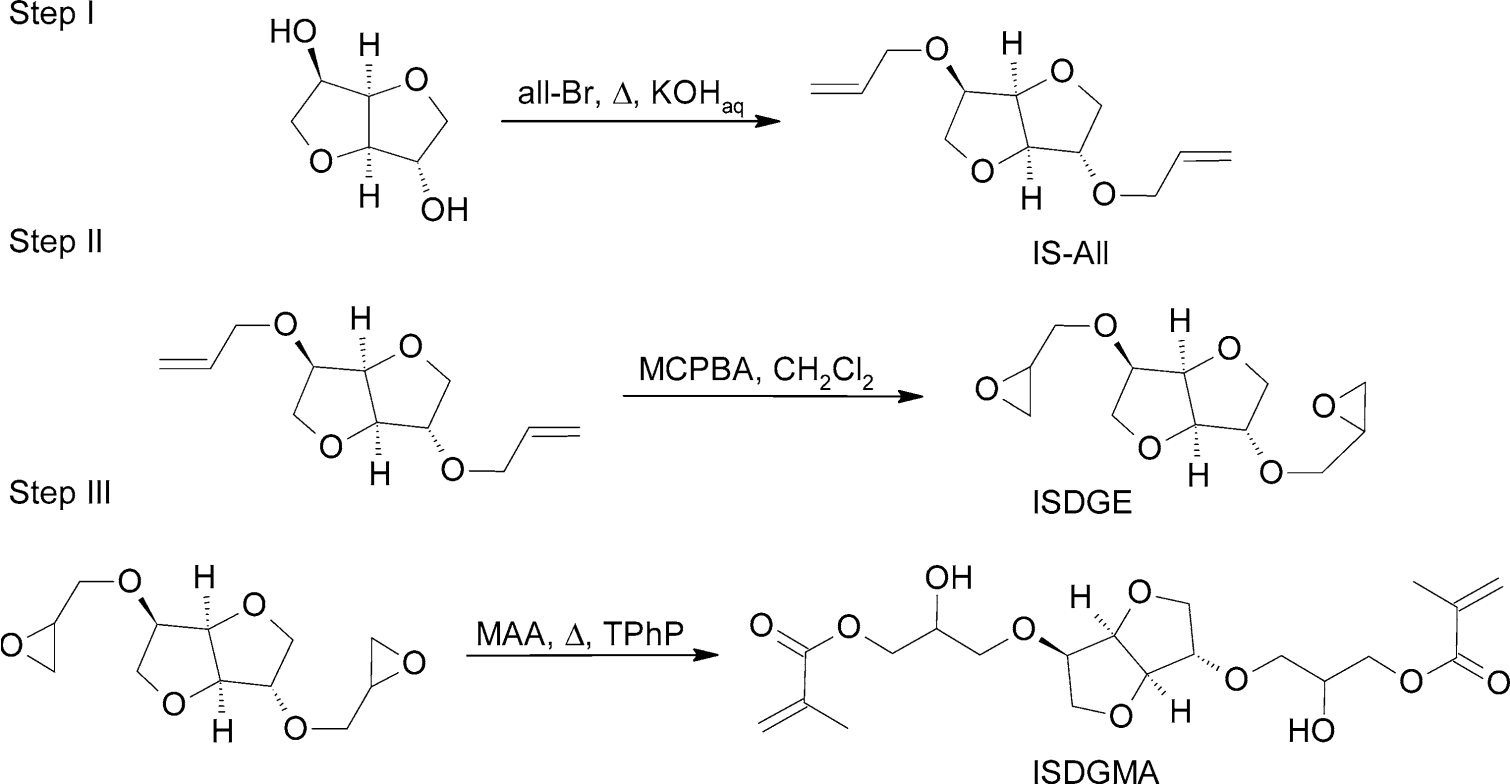
Scheme of the three-step synthesis of ISDGMA

The new isosorbide based dimethacrylate can be obtained with good yield of 80% in a three-step synthesis comprising etherification of a diol, subsequent oxidation and finally addition of methacrylic acid. Each of those steps requiring low cost materials and equipment leads to a product obtained in a high yield. Structure of resulting monomer that is shown in Fig. 1 was confirmed by means of nuclear magnetic resonance as well as infrared spectroscopy and MS spectrometry. Although, the catalytic addition of methacrylic acid to isosorbide diglycidyl ether proceeds with complete conversion of epoxy groups, the use of triphenylphosphine catalyst gives small amounts of a branched side product. This can be explained due to limited regioselectivity of the reaction. The isomeric structure of ISDGMA might be identified basing on a low intensity signal of methine proton at 5.03 ppm in ^1^H NMR spectrum. Comparison of basic physical properties of ISDGMA and BISGMA are shown in Table 1. Densities of both dimethacrylates are comparable, although in case of ISDGMA it is slightly higher. ISDGMA exhibits significantly lower viscosity than BISGMA, what can be related to weaker intermolecular forces of hydrogen bonds type. The new cycloaliphatic dimethacrylate viscosity is about 40 times lower at room temperature and 14 times lower at elevated temperature (40°C) than that of conventional aromatic monomer. Lower viscosity of ISDGMA may allow reducing the content of diluting comonomer below that applied in BISGMA based restorative systems.

**Table 1:** Basic physical properties of BISGMA and ISDGMA monomers

Properties	Monomer	
	BISGMA	ISDGMA
Molecular weight (*M*_w_) [g/mol]	512.6	430.4
Viscosity at 25°C (η) [Pa s]	462.3/25.9^a^	12.4/1.8^a^
Density at 25°C (*d*) [g/cm^3^]	1.16	1.21

^a^Additional measurement was made at 40°C

In case of homopolymer as well as copolymer based on new monomer double bond conversion is higher than that for reference materials and equal to 55 and 67% for ISDGMA and ISDGMA/TEGDMA composition respectively.

Phenomena like gelation or vitrification are responsible for limited conversion during polymerization in real systems. Nevertheless, high double bond conversion observed, indicates that the most of molecules present in the composition are attached with at least one end to the polymeric network. This diminishes unreacted monomer leaching and reduces eventual toxicity.

As can be seen in Table 2, ISDGMA has somewhat lower polymerization shrinkage than BISGMA in spite of higher conversion of double bonds although. In case of compositions, ISDGMA/TEGDMA shows higher shrinkage than BISGMA/TEGDMA but the difference is not significant. Results of DMA analysis show that glass temperatures of studied polymers range from 170 to 190°C. New polymers studied are characterized with high glass transition temperatures up to 189°C for poly(ISDGMA). This can be explained by the presence of bicyclic fragment of isosorbide as well as by high double bond conversion during photoplymerization, both responsible for high rigidity of polymer network.

**Table 2:** Properties of cured homo and copolymers

Properties	Polymers			
	Poly(BISGMA)	Poly(ISDGMA)	Poly(BISGMA-*co*-TEGDMA)	Poly(ISDGMA-*co*-TEGDMA)
Polymerization shrinkage (PS) [%]	13	10	8	11
Degree of double bonds conversion (DC) [%]	40	55	60	67
Glass transition temperature (*T*_g_) [deg C]	171	189	181	178
Water sorption (WS) [μg/mm^3^]	11.1 (1.4)	173.0 (2.1)	14.6 (1.7)	100.1 (0.9)

Standard deviations are presented in brackets

Results of determination of water sorption are also given in Table 2. For BISGMA based polymers water uptake is as follows: 11.1 μg/mm^3^ for poly(BISGMA) and 14.6 μg/mm^3^ for poly(BISGMA-*co*-TEGDMA), while for isosorbide based materials it is higher and ranges from 100.1 μg/mm^3^ for poly(ISDGMA-*co*-TEGDMA) up to 173.0 μg/mm^3^ for poly(ISDGMA). The undesired water sorption of isosorbide based polymers is much higher than that of conventional materials mainly because of hydroxyl groups present in the polymeric network. Despite having also hydroxyl groups, BISGMA based polymers exhibit lower water sorption due to the presence of hydrophobic aromatic rings and lower content of ether groups. In order to reduce water sorption prospective studies should focus on elimination of free hydroxyl groups for instance by means of alkylation or silanization. One can observe that poly(ISDGMA-*co*-TEGDMA) uptakes less water than the homopolymer. This may be due to the higher hydrophobicity of TEGDMA than ISDGMA.

**Fig. 2:**
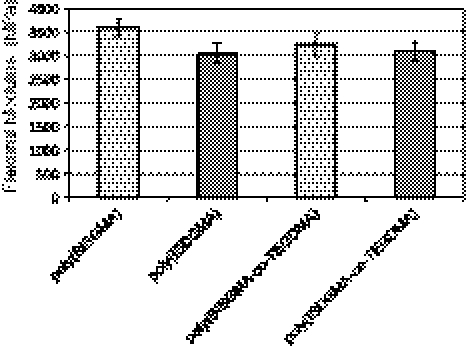
The average flexural modulus of investigated materials

**Fig. 3:**
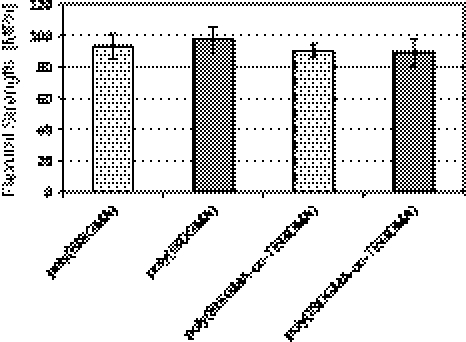
The average flexural strength of investigated materials

**Fig. 4:**
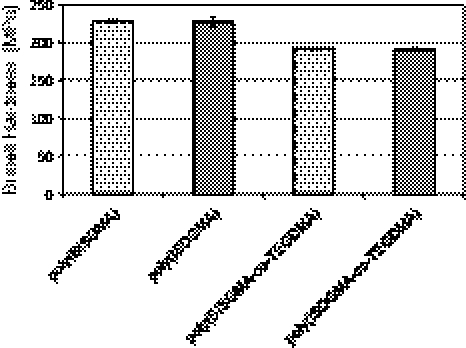
The average Brinell hardness of investigated materials

Selected mechanical properties of investigated materials are shown in Figs. 2, 3, 4. Polymers based on ISDGMA exhibit slightly lower flexural modulus than those based on BISGMA (Fig. 2). Flexural strength is comparable for all studied materials and ranges from 89 to 97 MPa. Moreover, both novel ISDGMA homopolymer and copolymer meet requirements of EN-ISO 4049.

Figure 4 presents results of Brinell hardness determination. It can be seen that hardness is equal for homopolymers and copolymers in each series. The addition of diluting comonomer results in reduction of hardness from 228 to 191 MPa. Introduction of isosorbide allowed obtaining polymers of good mechanical properties like high flexural strength and modulus as well as hardness comparable with conventional bisphenol A based materials.

In summary, the new dimethacrylic monomer ISDGMA may be considered as a potential substitute for commonly used but health uncertain BISGMA.

## Conclusions

A new dimethacrylate based on GRAS and bioderived alicyclic diol—isosorbide could be synthesized in a tree-step synthesis. It exhibits significantly lower viscosity than conventional BISGMA resin, thus the content of diluting comonomer can be reduced. Moreover, mechanical properties of ISDGMA polymers were found to be comparable to BISGMA reference materials. Degree of double bond conversion appears to be higher in case of isosorbide derivative. It is expected that presence of cycloaliphatic ether rings of isosorbide in the polymer network may provide a good stability in hydrolytic human oral environment. Its relatively high hydrophilicity however would require repeated estimation of mechanical properties of new materials being in equilibrium swelling state. Further research is going to be discussed in another paper.

## References

[1] Moszner N, Salz U (2001). New developments of polymeric dental composites. Prog Polym Sci.

[2] Ferracane JL (2011). Resin composite—state of the art. Dent Mater.

[3] Bowen L. US Patent 3066112;1962.

[4] Asmussen E, Peutzfeldt A (1998). Influence of UEDMA, BisGMA and TEGDMA on selected mechanical properties of experimental resin composites. Dent Mater.

[5] Chang MC (2010). The role of reactive oxygen species and hemeoxygenase-1 expression in the cytotoxicity, cell cycle alteration and apoptosis of dental pulp cells induced by BisGMA. Biomaterials.

[6] Yoshii E (1997). Cytotoxic effects of acrylates and methacrylates: relationships of monomer structures and cytotoxicity. J Biomed Mater Res.

[7] Theiling C, Tegtmeier Y, Leyhausen G, Geurtsen W (2000). Effects of BisGMA and TEGDMA on proliferation, migration and tenascin expression of human fibroblasts and keratinocytes. J Biomed Mater Res.

[8] Geurtsen W (2000). Biocompatibility of resin-modified filling materials. Crit Rev Oral Biol Med.

[9] Durner J, Dębiak M, Burkle A, Hickel R, Reichl FX (2011). Induction of DNA strand breaks by dental composite components compared to X-ray exposure in human gingival fibroblasts. Arch Toxicol.

[10] Chang MC (2009). The effect of BisGMA on cyclooxygenase-2 expression, PGE_2_ production and cytotoxicity via reactive oxygen species and MEK/ERK-dependent and independent pathways. Biomaterials.

[11] Guertsen W (1998). Substances released from dental resin composites and glass ionomer cements. Eur J Oral Sci.

[12] Podgórski M (2010). Synthesis and characterization of novel dimethacrylates of different chain lengths as possible dental resins. Dent Mater.

[13] Pereira SG, Osorio R, Toledano M, Nunes TG (2005). Evaluation of two Bis-GMA analogues as potential monomers diluents to improve the mechanical properties of light-cured composite resins. Dent Mater.

[14] Atai M, Nekoomanesh M, Hashemi SA, Amani S (2004). Physical and mechanical properties of an experimental dental composite based on a new monomer. Dent Mater.

[15] Sankarapandian M, Shobha HK, Kalachandra S, McGrath JE (1997). Characterization of some aromatic dimethacrylates for dental composite applications. J Mater Sci Mater Med.

[16] Rüttermann S, Dluzhaskaya I, Großsteinbeck C, Raab WHM, Janda R (2010). Impact on replacing BisGMA and TEGDMA by other commercially available monomers on the properties of resin-based composites. Dent Mater.

[17] Ge J, Trujillo M, Stansbury J (2005). Synthesis and photopolymerization of low shrinkage methacrylate monomers containing bulky substituent groups. Dent Mater.

[18] Chung CM (2002). Development of a new photocurable composite resin with reduced curing shrinkage. Dent Mater.

[19] Kawaguchi T, Lassila LVJ, Vallittu PK, Takahashi Y (2011). Mechanical properties of denture base resin cross-linked with methacrylated dendrimer. Dent Mater.

[20] Viljanen EK, Lassila LVJ, Skrifvars M, Villittu PK (2005). Degree of conversion and flexural propertiesof a dendrimer/methyl methacrylate copolymer: design of experiments and statistical screening. Dent Mater.

[21] Matinlinna JP, Lassila LVJ, Kangasniemi I, Yli-Urpo A, Vallittu PK (2005). Shear bond strength of Bis-GMA resin and methacrylated dendrimer resins on silanized titanium substrate. Dent Mater.

[22] Weinmann W, Thalacker C, Guggenberger R (2005). Siloranes in dental composites. Dent Mater.

[23] Ilie N, Hickel R (2006). Silorane-based dental composite: behavior and abilities. Dent Mater J.

[24] Lien W, Vandewalle KS (2010). Physical properties of a new silorane-based restorative system. Dent Mater.

[25] Malhotra SV, Kumar V, East A, Jaffe M (2007). Applications of corn-based chemistry. The bridge.

[26] Gohil RM (2009). Properties and strain hardening character of polyethylene terephthalate containing isosorbide. Polym Eng Sci.

[27] Stross P, Hemmer R (1991). 1,4:3,6-Dianhydrohexitols. Adv Carbohydr Chem Biochem.

